# Circulating neutrophil extracellular trap (NET)-forming ‘rogue’ neutrophil subset, immunotype [DEspR+CD11b+], mediate multi-organ failure in COVID-19 - *an observational study*

**DOI:** 10.21203/rs.3.rs-2479844/v1

**Published:** 2023-02-01

**Authors:** Victoria L.M. Herrera, Nicholas A. Bosch, Judith J. Lok, Mai Q. Nguyen, Kaitriona A. Lenae, Joanne T. deKay, Sergey V. Ryzhov, David B. Seder, Nelson Ruiz-Opazo, Allan J. Walkey

**Affiliations:** 1 Department of Medicine and Whitaker Cardiovascular Institute, Boston University Chobanian and Avedisian School of Medicine; 2 Section of Pulmonary and Critical Care Medicine, Department of Medicine, Boston University Chobanian and Avedisian School of Medicine; 3 Department of Mathematics and Statistics, Boston University; 4 Maine Health Institute for Research; 5 Department of Critical Care Services, Maine Medical Center

**Keywords:** COVID-19, multi-organ failure, NETs, neutrophil-subsets, DEspR, mediation analysis

## Abstract

**Background:**

Cumulative research show association of neutrophils and neutrophil extracellular traps (NETs) with poor outcomes in severe COVID-19. However, to date, no curative intent therapy has been identified to block neutrophil/NETs-mediated progression of multi-organ dysfunction. Because of emerging neutrophil heterogeneity, the study of subsets of circulating neutrophil-extracellular trap (NET)-forming neutrophils [NET+Ns] as mediators of multi-organ failure progression among patients with COVID-19 is critical to identification of therapeutic targets.

**Methods:**

We conducted a prospective observational study of circulating levels of CD11b+[NET+N] immunotyped for dual endothelin-1/signal peptide receptor, (DEspR±) expression by quantitative immunofluorescence-cytology and causal mediation analysis. In 36 consented adults hospitalized with mod-severe COVID-19, May to September 2020, we measured acute multi-organ failure via SOFA-scores and respiratory failure via SaO2/FiO2 (SF)-ratio at time points t1 (average 5.5 days from ICU/hospital admission) and t2 (the day before ICU-discharge or death), and ICU-free days at day28 (ICUFD). Circulating absolute neutrophil counts (ANC) and [NET+N] subset-specific counts were measured at t1. Spearman correlation and causal mediation analyses were conducted.

**Results:**

Spearman correlation analyses showed correlations of t1-SOFA with t2-SOFA (*rho r*_*S*_=0.80) and ICUFD (*r*_*S*_=−0.76); circulating DEspR+[NET+Ns] with t1-SOFA (*r*_*S*_= 0.71), t2-SOFA (*r*_*S*_ =0.62), and ICUFD (*r*_*S*_ =−0.63), and ANC with t1-SOFA (*r*_*S*_=0.71), and t2-SOFA (*r*_*S*_=0.61).

Causal mediation analysis identified DEspR+[NET+Ns] as mediator of 44.1% [95% CI:16.5,110.6] of the causal path between t1-SOFA (exposure) and t2-SOFA (outcome), with 46.9% [15.8,124.6] eliminated when DEspR+[NET+Ns] were theoretically reduced to zero. Concordantly, DEspR+[NET+Ns] mediated 47.1% [22.0,72.3%] of the t1-SOFA to ICUFD causal path, with 51.1% [22.8,80.4%] eliminated if DEspR+[NET+Ns] were reduced to zero. In patients with t1-SOFA >1, the indirect effect of a hypothetical treatment eliminating DEspR+[NET+Ns] projected a reduction of t2-SOFA by 0.98 [0.29,2.06] points and ICUFD by 3.0 [0.85,7.09] days. In contrast, there was no significant mediation of SF-ratio through DEspR+[NET+Ns], and no significant mediation of SOFA-score through ANC.

**Conclusions:**

Despite equivalent correlations, DEspR+[NET+Ns], but not ANC, mediated progression of multi-organ failure in acute COVID-19, and its hypothetical reduction is projected to improve ICUFD. These translational findings warrant further studies of DEspR+[NET+Ns] as potential patient-stratifier and actionable therapeutic target for multi-organ failure in COVID-19.

## Introduction

Activated neutrophils release neutrophil extracellular traps (NETs) - a DNA-weblike structure embedded with neutrophil microbicidal/cytotoxic proteases, enzymes, and decondensed histones - to entrap and eliminate pathogens [1, 2]. As a robust but non-targeted pathogen-killing defense mechanism, the microbicidal/cytotoxic components in NETs, like a double-edged sword, can also induce bystander “secondary” injury to vascular endothelia and adjacent cells. These injuries erode capillary-tissue barriers causing multi-organ dysfunction progressing to failure, even if the inciting infection is focal or decreasing [3, 4]. Cumulative research shows that increased NET-levels are associated with both severity of infection and risk for tissue injury, as seen in the association of increased NETs with COVID-19 severity [5, 6] and high mortality [7].

Preclinical studies show causal pathogenic mechanisms for NETs in SARS-CoV-2 virus infection, COVID-19. SARS-CoV-2 virus induces NET-formation in human healthy volunteer neutrophils, and the formed NETs cause injury in human epithelial and endothelial cells [7–9], including acute lung injury [10]. Concordantly, increased NET-levels have been implicated in all the clinical pathologies observed in the spectrum of severe COVID-19 multi-organ dysfunction including thromboses [11], parallel to observations in bacterial pneumonia and sepsis-induced models of acute lung injury or multi-organ failure (MOF) [12, 13]. The observed common pathogenic roles of excess NETs in secondary tissue injury, systemic micro-thrombosis or microvascular inflammation and occlusion, suggest reduction of NETs as a potential therapeutic approach to MOF, which to date remains without curative-intent therapy.

However, since NETs provide multiple key defense mechanisms against bacterial infections [2], sepsis [14] and viral infections [15], therapeutic approaches to block NETs-mediated secondary “bystander” tissue injury must target dysregulated NET-formation, but spare homeostatic regulated NET-formation. Given neutrophil heterogeneity and multiple mechanisms of NET-formation [16, 17], identification of dysregulated “rogue” NET-forming neutrophil subsets/subtypes that escape normal NET-clearance, accumulate, and contribute to multi-organ failure could be key to much-needed targeted therapies for severe COVID-19.

Our recent studies have identified DEspR+CD11b+ neutrophils (DEspR+[Ns] from hereon), as a dysregulated “rogue” neutrophil-subset capable of NET-formation while in the circulation, extended survival, low-clearance, enhanced adhesion, and association with severity measures and mortality in COVID-19 acute respiratory distress syndrome (ARDS) [18–20]. Here, we test whether the putative “rogue” CD11b+DEspR+ NET-forming neutrophil-subset, DEspR+[NET+N], mediates the worsening of multi-organ dysfunction (as measured by the Sequential Organ Failure Assessment [SOFA]-score) [21] and poor clinical outcomes (as measured by intensive care unit free days [ICUFD]) [22] in severe COVID-19 using causal mediation analysis. Identification of a rogue [NET+N] subset that mediates progression of multi-organ dysfunction in severe COVID-19 patient samples has the potential to identify a much-needed therapeutic target and/or biomarker. The combinatorial use of direct analysis of patient neutrophils and causal inference mediation statistics has the potential to validate a translational approach to overcoming low translatability of animal models in ARDS-multi-organ failure regardless of etiology.

## Methods

### Study design and Participants.

Procedures followed were in accordance with the ethical standards of the responsible committee on human experimentation (institutional or regional) and with the Helsinki Declaration of 1975, as most recently amended (https://www.wma.net/policies-post/wma-declaration-of-helsinki-ethical-principles-for-medical-research-involving-human-subjects/).

A combined 2-site analysis of NET-forming neutrophil subsets from two independent prospective observational study cohorts previously characterized for ‘rogue’ neutrophils and NETosis [18, 19]. At Boston Medical Center (BMC), the protocol study number is H-36744, study title: Humanized anti-DEspR antibody therapy for Acute Lung Injury (ALI/Acute Respiratory Distress Syndrome), and approval by Boston University’s Institutional Review Board on 12-01-2019. At Maine Medical Center (MMC), the protocol study number is 1598969–16, with study title “IT-19 Identification of molecular treatment targets for COVID-19”, and approval by Maine Medical Center’s Institutional Review Board on 5/8/2020. Clinical data and blood sample collections followed IRB approved protocols, (Supplemental Methods). Informed consent was obtained from the patient when able, or when unable, from the patient’s legally authorized representative (LAR). LAR-informed consents were obtained by phone using an IRB-approved informed consent-by-phone at BMC, or electronically at MMC, to minimize viral exposure. Clinical parameters of severity were obtained: non-neurologic SOFA score as a measure of multi-organ dysfunction and SaO_2_/FiO_2_ (SF)-ratio as a measure of respiratory distress/failure, and ICU-free days at day 28, with competing risk of death −1 (ICUFD) (22) as a summation outcome measure. Clinical measures were taken from two time points: timepoint-1 (t1) after informed consent after COVID19 diagnosis verification upon admission to the hospital or ICU, average 5.5 days, and timepoint-2 (t2): the day before ICU-death or ICU/hospital discharge. CBC-differential and blood samples for immunofluorescence cytology were obtained at timepoint-t1.

### Rigor and Reproducibility.

Rigor was ascertained via compartmentalized blinding in research procedures. Clinical data collection was done blinded to determination and quantitation of [NET+N] subsets and vice versa. Quantitative imaging of [NET+N] subsets was done independently by a 3^rd^ party blinded to clinical data. Causal mediation analysis was performed by researchers not involved in data collection. (Supplemental Methods for detail)

### Immunofluorescence cytology (IFC): immunofluorescence (IF)-staining of patient ‘blood smears’.

Cytology slides were prepared directly from whole blood within 1hr from blood draw in EDTA-anticoagulated samples (BMC) [19, 23] and within 1–3hrs from blood draw from acid-citrate dextrose samples (MMC) in order to preserve, hence detect circulating neutrophils with fragile DNA-webs/strands, as first observed in activated NET-forming neutrophils *ex vivo* by Brinkmann et al 2004 [2]. To directly measure subset-specific [NET+Ns] in the circulation in a clinically feasible and safe way in COVID-19 patients, cytology slides were fixed in −20°C 100% methanol prior to immunotyping for: 1] CD11b expression, an established marker of activated neutrophils in COVID-19 capable of NET-formation [2], and 2] DEspR expression expressed on rogue neutrophil-subset with extended lifespan and associated with severity in non-COVID-19 ARDS and COVID-19-ARDS [19], 3] 4′,6-diamidino-2-phenylindole (DAPI) staining for detection of DNA strands/webs still attached to CD11b+ neutrophils, and 4] citrullinated Histone 3 as marker of decondensed histones characteristic of NET-formation [2, 24]. (Supplemental methods for detail)

### Semi-automated Quantitation of [NET+Ns] subsets.

Third party Nikon Imaging Laboratory (Cambridge MA) performed quantitative imaging analysis blinded to patient information for automated unbiased detection and quantitation of NET-forming DNA-extruding neutrophils by measuring the circularity index using a standard shape analysis formula 4pi (area/perimeter^2^) validated earlier [19]. Quantitative analysis of subsets of NET-forming neutrophils with molecular markers for CD11b± and DEspR± and DNA was performed via automated determination of fluorescence intensities of NET-forming neutrophils identified by circularity index < 0.8. (Supplemental Methods for detail)

### Determination of measures of [NET+Ns].

From the IF-cytology analysis and quantitation of [NET+Ns], the % of DEspR+ vs DEspR(−) [NET+Ns] was determined from the total NET+Ns detected with circularity index <0.8 indicating extruded DNA. Data were exported to a CSV file, final scoring was completed in Excel. The number of DEspR+ *vs* DEspR(−) [NET+Ns] was calculated by multiplying the % DEspR+[NET+Ns] × ANC obtained on the same day complete blood count (CBC)-differential.

### Statistical Analyses.

Descriptive statistics and Spearman rank correlation analysis were performed using (PRISM 9.4, GraphPad). Power analyses were performed using SigmaStat 11.0.

#### Spearman correlation analysis.

To select putative mediators for causal mediation analysis, we determined linear relationships of [NET+N] subset-specific levels with clinical severity measures relevant to the progression of multi-organ failure, we performed Spearman correlation analysis. For putative comparator mediators, we also analyzed DEspR+CD11b+ neutrophil counts, neutrophil-to-lymphocyte ratio (NLR), and ANC. Spearman correlation coefficient (*r*_*S*_) greater than *r*_*S*_ .46, for n = 36 subjects, was estimated to give power 0.8, at alpha 0.05. We performed Bonferroni correction of Spearman correlation *P* values: *P* × the number of hypotheses tested, in our study n = 7 hypotheses.

#### Causal Mediation analysis (CMA)

Causal Mediation analysis (CMA) was performed using R (version 4.2.1) and package regmedint [25]. Causal mediation analysis seeks to disentangle relationships between three or more variables [26–28] where some or all of the effect of an exposure (A) on an outcome (Y) is mediated by a mediator of interest (M). Causal mediation analysis quantifies the direct effect of A on Y and the indirect effect of A on Y through the mediator M. In addition, causal mediation analysis accounts for interaction between the exposure and mediator such that the strength of the association of the indirect mediated pathway may depend on the value of the exposure and mediator variables.

Here, we examined DEspR+CD11b+ NET+Ns as potential mediators of 1) progressive multi-organ dysfunction (t1-SOFA score to t2-SOFA score), 2) progressive pulmonary specific organ dysfunction (t1-SF to t2-SF), and 3) t1-SOFA and t1-SF to length of hospital/ICU stay accounting for the competing risk of death (ICU free days [ICUFD]). We considered both mediator and interaction effects between t1 variables and DEspR+[NET+N] counts. For each causal pathway the primary estimand of interest was the percent mediated (i.e, the percent of the effect of the exposure on the outcome mediated by DEspR+[NET+N] counts) and the second estimand of interest was the percent eliminated (i.e., the percent of the effect of the exposure on the outcome that would be removed if DEspR+[NET+N] counts were reduced to zero). Because the exposures were continuous variables, we modeled the mediation effects of DEspR+[NET+N] counts due to a moderate change in the exposure variable (i.e., from its first quartile value to its third quartile value). The pure natural direct effect (the direct effect of exposure on outcome if the mediator [NET+Ns] is set at the value it would naturally take when exposure is at its reference value), the total natural direct effect (the direct effect of exposure on outcome accounting for exposure and mediator interaction), and the total effect (total effect of exposure on outcome through direct and indirect pathways) were also reported. As comparator, we also tested absolute neutrophil counts (ANC) as a mediator of t1-SOFA to t2-SOFA and t1-SOFA to ICUFD. We used bootstrapping with 10,000 replicates to calculate 95% confidence intervals.

#### Analysis of effects of a hypothetical treatment reducing DEspR+[NET+Ns] as mediator.

We estimated the indirect effect on t2-SOFA, ICUFD, and t2-SF ratio mediated by DEspR+[NET+Ns] of a hypothetical treatment that would eliminate DEspR+[NET+Ns] as described by Lok and Bosch [29], which showed that a treatment effect on the mediator can be combined with off-treatment mediator and outcome data to estimate a causal indirect effect (but not a total or direct effect). To estimate the indirect effect on t2-SOFA and ICUFD mediated by DEspR+[NET+Ns] of a hypothetical treatment that would eliminate DEspR+[NET+Ns] (ie, zero-level), we included t1-SOFA ≥ 2 as a pre-treatment common cause of DEspR+[NET+Ns] and t2-SOFA/ICUFD, and also included t1-SOFA ≥ 4 as a second pre-treatment causal variable. For the t2-SF ratio, we included the t1-SF ratio as a pre-treatment common cause. For these analyses we modified existing SAS macros (29), for a continuous outcome modeled with linear regression similar to regmedint [25].

## RESULTS

### Patient characteristics

COVID-19 subjects were enrolled from May to September 2020 prior to vaccinations or anti-viral therapies at Boston Medical Center and at Maine Medical Center. These subjects were analyzed independently earlier for “rogue” DEspR+CD11b+ neutrophils using lab-specific flow cytometry experimental protocols respectively [18, 19]. Here, combined 2-site characteristics are summarized in Table 1. Of the 36 subjects, mean age 63.6 years, 67% were males, 67% met the Berlin Definition for ARDS (both male and female), 81% had corticosteroid therapy, 31% were diagnosed with clinical thrombosis, and 6% were placed on hemodialysis (Table 1). Among the 36 subjects, the median t1-SOFA-score was 2.5 (interquartile range [IQR] 1–6.25; range 0–12) and median t2-SOFA-score was 1 (IQR 0, 4.25; range 0–11). Median SF-ratios were 242.5 (IQR 135.8–346.2; range 77–457) at t1 and 334.5 (IQR 243.0–439.2; range 87.0–471.0) at t2. Now, we present the combined 2-site study of putative subsets of circulating NET-forming neutrophils analyzed in a common facility using identical protocols for immunotyping and semi-automated quantitation.

### Circulating NET-forming neutrophil (NET+N) subsets in mod-severe acute COVID-19

**Figure 1.**
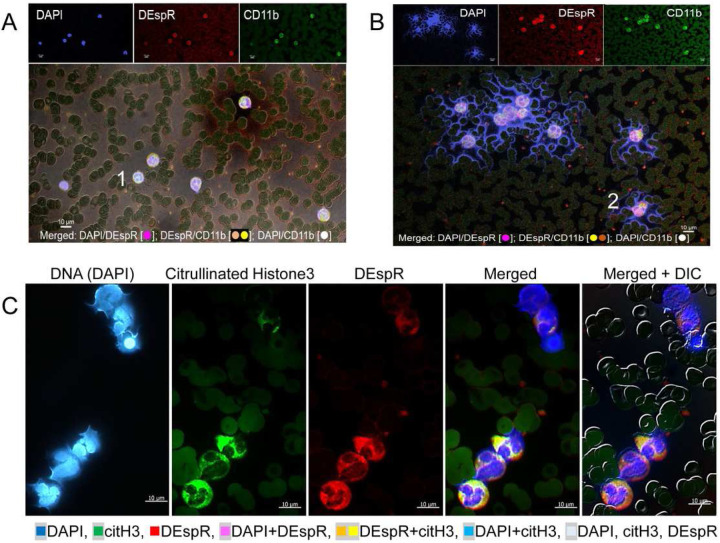
Representative images of differential levels of circulating NET+Ns by immunofluorescence-cytology (IFC). **(A-B)** Representative IFC confocal microscopy image [z-stack through nucleus] showing minimal to no DEspR+CD11b+[NET+Ns] with extruded DNA (blue) in a (A) COVID-19 survivor, and (B) increased DEspR+[NET+Ns] in COVID-19 non-survivor. Bar 10 μm; → DEspR+CD11b+ microvesicles. **(C)** Representative IFC images of DEspR+[NET+Ns] showing from left to right: DAPI-DNA staining of decondensing nuclei in various stages of extrusion of DAPI-stained DNA, citrullinated histone-3 (citH3) expression with varying intensities, neutrophil DEspR+ expression, merged image, and merged image with differential interference contrast (DIC) superimposed. Bar = 10 microns.

**Figure 2.**
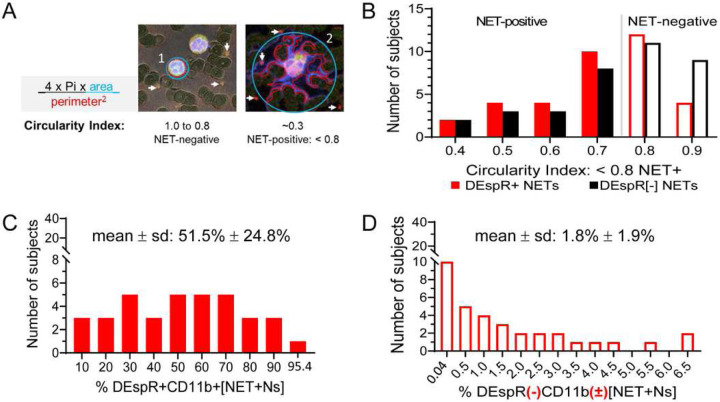
Comparative quantitative analysis of circulating NET+Ns using automated shape analysis in COVID-19 peripheral blood smears. **(A)** Diagram of circularity index determination and corresponding frequency graph of the spread of circularity indices calculated by automated shape-analysis algorithm with designation of < 0.8 as cut-off for assignment of NET-forming neutrophils or [NET+Ns]. **(B)** Frequency curve of number of COVID-19 subjects (n = 36) with DEspR+CD11b+[NET+Ns]: median 53.4% (IQR: 28.2 – 68.5%), mean ± sd: 51.5% ± 24.8%. **(C)** Frequency curve of number of COVID-19 subjects (n = 36) with DEspR(−)CD11b(±) [NET+Ns]: median 1.0% (IQR: 0.2 – 3.1%); mean ± sd: 1.8% ± 1.9%.

IFC-immunostaining of patient blood smears detected differential (CD11b±DEspR±) subset-specific levels of circulating NET+Ns in patients with severe COVID-19 (Table 1, Fig. 1–2). Representative IFC-images of [NET+Ns] show differences in a COVID-19 patient who survived (Fig. 1A) compared to a patient with multi-organ failure who died (Fig. 1B). IFC-immunostaining confirms NET-forming neutrophil features: [2, 24] extruded DNA, nuclear decondensation, histone-3 citrullination (citH3), plasma-membrane changes, neutrophil-derived microvesicles, (Fig. 1B–C).

Semi-automated quantitation of circulating [NET+N] subsets based on shape analysis of circularity (Fig. 2A), we detected both DEspR+ and DEspR(−) [NET+N] subsets with differential frequencies (Fig. 2B) in our COVID-19 cohort. DEspR+[NET+Ns] comprised 51.5% ± 24.8% (mean ± SD) of circulating neutrophils (Fig. 2C), compared with 1.8% ± 1.9% (mean ± SD) DEspR(−) [NET+Ns] (Fig. 2D). Frequency distribution also showed a wide range of % DEspR+[NET+Ns] among all neutrophils in COVID-19 patients (Fig. 2C–D). Because of variations in absolute neutrophil counts, we derived the absolute number of DEspR+[NET+Ns] × 10^3^ (K) per μL blood for subsequent analyses (Table 1).

### Correlation of circulating DEspR+[NET+N] levels and severity measures

Spearman correlation analyses detected strong Spearman correlation coefficients (*r*_*S*_) ranging from 0.62–0.71 for DEspR+[NET+N] counts with clinical indicators of severity of multi-organ dysfunction at both time points (Table 2, Supplemental Fig. S1 for scatter plots). Concordantly, strong negative correlation of DEspR+[NET+N] counts and ICUFD was observed (*r*_*S*_ = −0.63). Strong correlations of NLR and absolute neutrophil counts (ANC) with SOFA and SF-ratio at both timepoints were also observed (Table 2). Other subsets of NET-forming neutrophils: DEspR(−) [NET+Ns] (range *r*_*S*_ 0.45 – 0.6), DEspR+[Ns] that are not NET-forming (range *r*_*S*_ 0.44 – 0.60) exhibited weaker correlation coefficients (Table 2).

### Causal Mediation Analysis of circulating DEspR+[NET+N] levels

**Figure 3.**
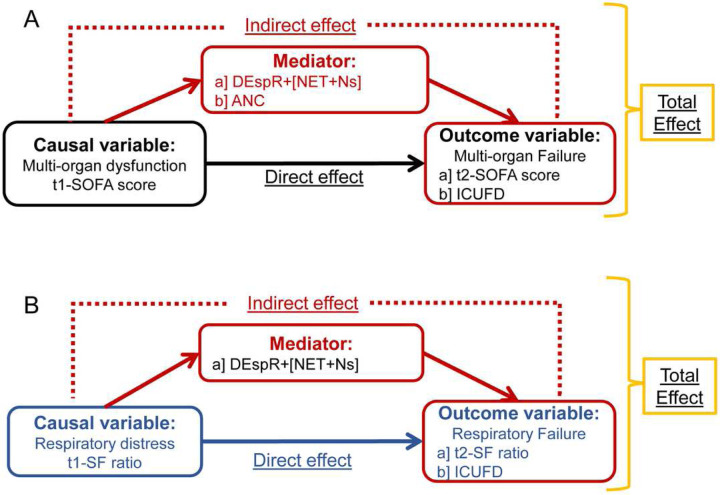
Directed acylic graphs of causal hypotheses for mediation analysis of hypothesized mediators: DEspR+[NET+Ns] or comparator ANC in COVID-19. **(A)** Hypothesis-1: DEspR+[NET+Ns] mediate progression of multi-organ dysfunction causal path between t1 (t1-SOFA score) to multi-organ failure (t-2 SOFA score) or poor clinical outcomes (ICUFD). Alternative mediator tested: ANC, absolute neutrophil counts. **(B)** Hypothesis-2: DEspR+[NET+Ns] mediate progression of respiratory distress at t1 (t1-SF ratio) to respiratory failure at t2 (t2-SF) or poor clinical outcomes (ICUFD). SOFA-score, obtained without neurological component; ICUFD, ICU-free days by day 28 with 0 for patients in the ICU > 28 days and competing risk of death [−1].

Based on preclinical causal relationships of SARS CoV2, NET-formation, and injury to endothelial-tissue barrier in different vital organs contributing to multi-organ failure and supported by corresponding correlation analyses depicting linear relationships (Table 2, Supplemental Fig. S1), we tested causal path hypotheses for progression of multi-organ failure (Fig. 3-A) and progression of respiratory failure (Fig. 3-B). As shown in Table 3, Mediation Analysis estimated that 44.1% [95% CI: 16.5, 110.6] of the relationship between t1-SOFA (modeling a change in t1-SOFA from its first quartile value [1 point] to its third quartile value [6.25 points]) and t2-SOFA (outcome) was mediated by DEspR+CD11b+ NET+Ns and that 46.9% [95% CI: 15.8, 124.6] of the effect of t1-SOFA on t2-SOFA would be eliminated by reducing DEspR+CD11b+ NET+Ns to 0. Similarly, in the proposed causal pathway between t-1 SOFA and ICUFD, 47.1% [22.0, 72.3%] of the relationship was mediated by DEspR+CD11b+ NET+Ns and 51.1% [95% CI: 22.8, 80.4] of the relationship between t1-SOFA and ICUFD would be eliminated by reducing DEspR+CD11b+ NET+Ns to 0. Concordantly, analysis of a hypothetical therapeutic that would reduce DEspR+[NET+Ns] to zero in patients with t1-SOFA ≥ 2 predicted a decrease in t2-SOFA of 0.98 [95% CI: 0.29, 2.06] points as compared with no therapeutic, and for patients with t1-SOFA ≥ 4, a decrease in t2-SOFA of 1.4 [95% CI: 0.47, 3.05] points (Table 3), indicative of a decrease in progression of multi-organ failure.

In contrast, mediation analysis for progression to acute respiratory failure (Fig. 3) showed no significant mediation by DEspR+[NET+Ns] between t1-SF and t2-SF, and between t1-SF and ICUFD (Table 3). Additionally, despite strong correlations with t2-SOFA and ICUFD (Table 2), there was no significant evidence of mediation by ANC on both causal path hypotheses for progression of multi-organ failure (Fig. 3-A): from t1-SOFA to t2-SOFA and from t1-SOFA to ICUFD (Table 3).

## Discussion

Premised upon preclinical studies detecting a causal role of SARS CoV2 on NET-formation and NET-forming neutrophils on capillary-tissue barriers, [8, 9] this prospective study of patients with acute COVID-19 identified DEspR+[NET+Ns] as a mediator of multi-organ failure progression. These observations are supported by the causal role of NETs in direct endothelial cell injury demonstrated in *ex vivo* studies [8–10, 30], and the causal role of DEspR in extending neutrophil lifespan [20]. We note that while causal mediation analysis has been previously used to evaluate inflammatory mediators of the effect of obesity on risk for mortality in COVID-19 [31], and soluble RAGE receptor levels and angiopoietin-2-levels in sepsis-related ARDS [32, 33], here we report causal mediation analysis of NET-forming neutrophil subsets.

The differential mediation effect profiles for DEspR+[NET+Ns] compared with ANC, despite similarly strong correlations with multi-organ failure outcomes, highlight the emerging role of NET+Ns as a central mechanism for neutrophil-mediated secondary tissue injury and/or immunothrombosis in multi-organ failure in COVID-19 [7, 34–36]. Likewise, the differential mediation of worse SOFA score and ICUFD – but not of worse SF-ratio – by DEspR+[NET+Ns] – indicates specificity of causal effect estimates of mediation. This differential mediation supports the hypothesis that the low SF-ratio in COVID-19 is caused by the direct damage of respiratory epithelia infected with the SARS CoV2 virus [37], rather than by neutrophil-mediated tissue injury of indirect ARDS [38]. Taken together, concordance of our findings supports the pathogenic role of circulating NET+Ns in direct endothelial injury and microcirculation compromise in the progression of secondary multi-organ failure in COVID-19.

In the analysis of a hypothetical treatment that eliminates DEspR+[NET+Ns] among patients with a SOFA score of 2 or more, elimination of DEspR+[NET+Ns] was associated with an indirect effect of a 1-point decrease in subsequent SOFA score, which for patients with COVID-19 ARDS, would translate approximately to a 15% absolute risk reduction (ARR) in death [39]. Similarly, elimination of DEspR+[NET+Ns] was associated with a 3-day increase in ICUFD. These results suggest that a novel treatment that eliminates DEspR+[NET+Ns] could potentially reduce mortality in severe COVID-19 with an effect estimate at least as strong as that of corticosteroids [40, 41].

Notably, preclinical studies support the feasibility of this therapeutic hypothesis as the anti-DEspR antibody induces apoptosis in DEspR+ neutrophils observed on live cell imaging of macaque neutrophils and promotes neutrophil apoptosis without worsening elevated myeloperoxidase and complement activation levels, as observed in an *ex vivo* experimental system testing ARDS patient whole blood samples [19]. Intuitively, the induction of apoptosis in circulating “rogue” [DEspR+CD11b+] neutrophils will pre-empt DEspR+[NET+Ns], but spare DEspR(−)[NET+Ns] and DEspR(−)neutrophil roles in key defense mechanisms against pathogens, thus likely avoiding the increases in infection risk seen in total neutrophil/NET+N inhibition or depletion.

Concordantly, data also reveal new concepts. The strong correlation of absolute number of DEspR+[NET+Ns] (range r_S_ 0.62 – 0.71) compared with an alternative parameter, the % of DEspR+[NET+Ns] (range r_S_ 0.27 – 0.35), suggests a circulating “[NET+N]-burden” hypothesis in progression of multi-organ failure, parallel to tumor cell-burden in cancer progression [42]. The strong correlation of DEspR+[NET+Ns] with SOFA-score we report here as compared with that previously observed for plasma NET-biomarker MPO-DNA complexes [7] indicates the advantage of quantitative IF-cytology (qIFC) subset-specific analysis of circulating DEspR+[NET+Ns], and that qIFC of whole blood smears provides a pathogenically relevant measure of circulating subset-specific NET-forming neutrophil levels. This method opens the door to identification and comparative analysis of other molecular subsets of NET-forming neutrophils in COVID-19 as potential causal intermediate(s) in different critical care pathologies wherein NETs are implicated. Lastly, if borne out in development of novel therapeutics, causal mediation analyses may be a useful tool to translate preclinical causality and efficacy to validated therapeutic hypotheses for clinical trial and address the unmet need arising from cumulative low translatability of preclinical animal models of ALI/ARDS to clinical trial efficacy. This potential is supported by an earlier study showing that causal mediation analysis can identify the promising treatment among different candidates for further testing in randomized clinical trials [29].

### Limitations of study:

Our small cohort size may be underpowered to detect weaker associations. This observational study was done during the first phase of COVID-19 without vaccination or anti-viral therapies available; thus, how these therapies may modify the relationships between organ dysfunction and NETs is unclear. Since NET levels vary with time and t1 was not the same for all patients due to informed consent issues, the study design may have introduced t1 noise/variation that decreases power.

### Conclusions and Clinical Implications:

In this prospective pilot study, Causal Mediation Analysis detected DEspR+[NET+N] subset as a mediator of progression of multi-organ-dysfunction in COVID-19 and the hypothetical reduction of DEspR+[NET+N] subset support the therapeutic hypothesis that prevention or reduction of DEspR+[NET+Ns] has the potential to reduce progression to multi-organ failure in severe acute COVID-19. Altogether, data provide translational milestones in support of further studies to advance DEspR+[NET+Ns] as a much-needed potential biomarker for patient stratification and therapeutic target for multi-organ failure in severe acute COVID-19.

## Data Availability

Data are available from corresponding author upon reasonable request.
